# Torin1-mediated TOR kinase inhibition reduces Wee1 levels and advances mitotic commitment in fission yeast and HeLa cells

**DOI:** 10.1242/jcs.146373

**Published:** 2014-03-15

**Authors:** Jane Atkin, Lenka Halova, Jennifer Ferguson, James R. Hitchin, Agata Lichawska-Cieslar, Allan M. Jordan, Jonathon Pines, Claudia Wellbrock, Janni Petersen

**Affiliations:** 1 Faculty of Life Sciences, University of Manchester, Michael Smith Building, Manchester M13 9PT, UK; 2 Cancer Research UK Drug Discovery Unit, Paterson Institute for Cancer Research, University of Manchester, Wilmslow Road, Manchester M20 4BX, UK; 3 The Gurdon Institute and Department of Zoology, Tennis Court Road, Cambridge CB2 1QN, UK

**Keywords:** HeLa, *S. pombe*, TOR, Torin1, Wee1

## Abstract

The target of rapamycin (TOR) kinase regulates cell growth and division. Rapamycin only inhibits a subset of TOR activities. Here we show that in contrast to the mild impact of rapamycin on cell division, blocking the catalytic site of TOR with the Torin1 inhibitor completely arrests growth without cell death in *Schizosaccharomyces pombe*. A mutation of the Tor2 glycine residue (G2040D) that lies adjacent to the key Torin-interacting tryptophan provides Torin1 resistance, confirming the specificity of Torin1 for TOR. Using this mutation, we show that Torin1 advanced mitotic onset before inducing growth arrest. In contrast to TOR inhibition with rapamycin, regulation by either Wee1 or Cdc25 was sufficient for this Torin1-induced advanced mitosis. Torin1 promoted a Polo and Cdr2 kinase-controlled drop in Wee1 levels. Experiments in human cell lines recapitulated these yeast observations: mammalian TOR (mTOR) was inhibited by Torin1, Wee1 levels declined and mitotic commitment was advanced in HeLa cells. Thus, the regulation of the mitotic inhibitor Wee1 by TOR signalling is a conserved mechanism that helps to couple cell cycle and growth controls.

## INTRODUCTION

Cells regulate growth, metabolism and proliferation through target of rapamycin (TOR) kinase signalling. Fission yeast *Schizosaccharomyces pombe* contains two TOR kinases: the non-essential Tor1 and the essential Tor2 (Weisman and Choder, 2001). TOR kinases participate in at least two distinct protein complexes: TORC1 (mainly containing Tor2) and TORC2 (predominantly containing Tor1) (Alvarez and Moreno, 2006; Hayashi et al., 2007; Matsuo et al., 2007). It is established that rapamycin inhibits a subset of TOR activities in TORC1 complexes. In *Saccharomyces cerevisiae* rapamycin promotes growth arrest (Barbet et al., 1996); however, it does not show the same effect in either *S. pombe* or some mammalian cells (Neshat et al., 2001; Pedersen et al., 1997; Weisman et al., 1997). In contrast, treatment of mammalian cells with ATP-analogues that target the kinase domain of mTOR, such as Torin1 (Thoreen et al., 2009), mimics the impact of rapamycin treatment in budding yeast, in that they induce autophagy, reduce protein synthesis and arrest cell cycle progression in G1 with a reduced cell size (Thoreen et al., 2009). These effects of Torin1 established that there are rapamycin-resistant roles for mTORC1 that are essential for growth and proliferation. Torin1 interacts with tryptophan-2239 in the catalytic, active site of mTOR kinase (Yang et al., 2013). Crucially, this residue is absent in other kinases, including the mTOR-related phosphoinositide 3-kinases (PI3Ks).

Here, we describe the isolation of a *tor2* mutation that maps to a conserved glycine located next to the key tryptophan (W2239 of mTOR) that directly interacts with Torin. This mutation conferred resistance to Torin1 and functionally validated the specificity of Torin1 for TOR kinases. We have exploited this Torin1-resistant mutation to show that complete TORC1 inhibition advanced mitotic commitment. Torin1 treatment reduced the levels of the mitotic inhibitor Wee1. Experiments in human cell lines recapitulated these yeast observations: Wee1 levels decreased and mitotic commitment advanced when HeLa mTOR was inhibited by Torin1. These findings provide novel insight into the mechanisms by which inhibition of TOR activity impacts upon mitosis and cell division.

## RESULTS

### Growth of *S. pombe* is inhibited without cell death or G1 arrest following Torin1-induced TOR inhibition

**Fig. 1.**
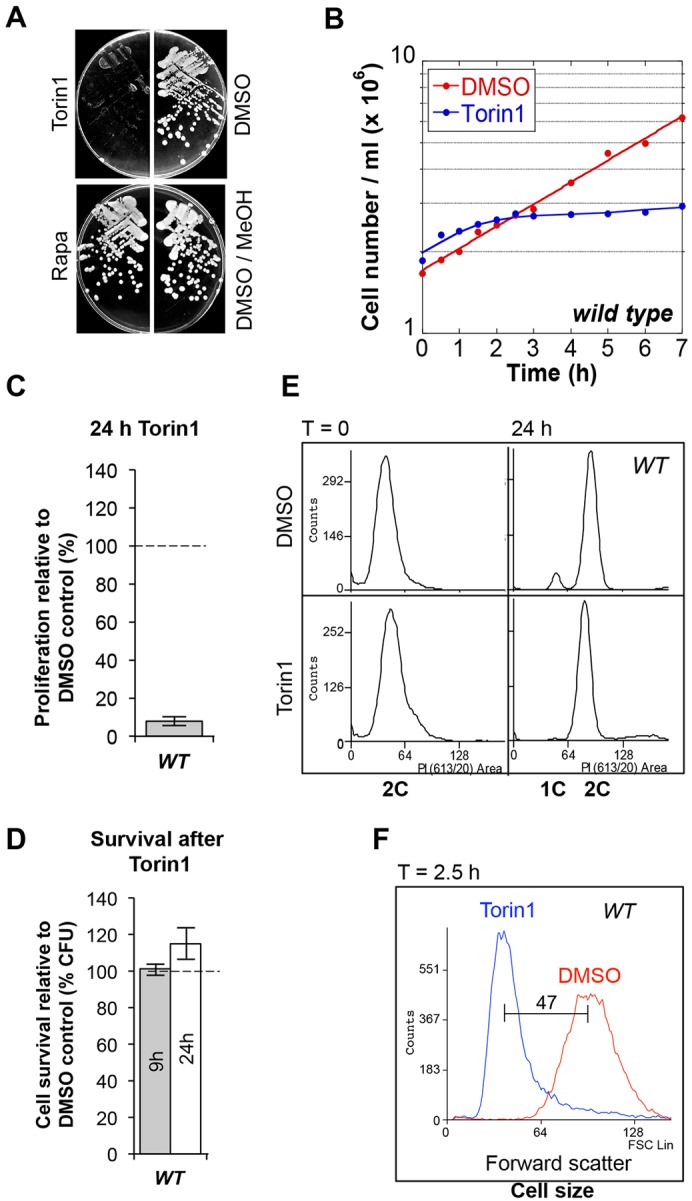
**Growth of *S. pombe* is inhibited without cell death or G1 arrest following inhibition of TOR signalling by Torin1.** (A) Wild-type cells grown on EMMG plates containing 25 µM Torin1, 300 ng/ml rapamycin or solvent. MeOH, methanol. (B-F) Liquid cultures were treated with 25 µM Torin1 or DMSO. (B) Cell number was measured and proliferation relative to vehicle calculated after 24 hours (C). (D) 500 cells were spread on YES plates and colony-forming units counted and shown relative to vehicle-treated cultures. (E) DNA content was analysed by flow cytometry. (F) Cell size was determined by forward-scatter flow cytometry.

We wanted to exploit TOR inhibition by Torin1 to further characterise TOR signalling in the model eukaryote *S. pombe*. A recent study has shown that a low concentration of Torin1 (0.2 µM) inhibits TORC1; however, no growth arrest of wild-type (wt) cells is observed (Ma et al., 2013). As the *tor2^+^* (TORC1 complex) gene of fission yeast is essential (Weisman and Choder, 2001), TOR inhibition would be expected to halt growth and proliferation. The ATP analogue (25 µM) did indeed inhibit growth of wild-type cells on minimal solid media or in liquid cultures (Fig. 1A–C). On rich media (YES), the growth of wt cells was inhibited by 5 µM Torin1 (data not shown). Incubation with the drug for 24 hours reduced proliferation to less than 10% of vehicle-treated control cultures (Fig. 1C). As previously reported, rapamycin had only a marginal impact on growth (Fig. 1A) (Weisman et al., 1997). To address whether Torin1 was promoting cell death, cells were treated with Torin1 for 9 or 24 hours and spread on plates containing rich medium without Torin1 to assess viability. Torin1-treated and vehicle-treated control cultures gave similar numbers of colony forming units (CFU) (Fig. 1D), indicating that cells resumed growth following Torin1 withdrawal. In other words, Torin1 inhibition did not induce cell death. We therefore asked whether the growth arrest arose from cell cycle arrest in G1, as seen in mammalian cells (Thoreen et al., 2009) and in fission yeast following Tor2 inhibition (Matsuo et al., 2007; Uritani et al., 2006). Flow cytometric analysis demonstrated that, in contrast to mammalian cells, wild-type fission yeast cells did not arrest in G1 after incubation with the drug for up to 24 hours (Fig. 1E). Importantly, despite this lack of a G1 arrest, cell size was reduced following TOR inhibition (Fig. 1F; Fig. 4A). These data demonstrated that Torin1 inhibited *S. pombe* growth without inducing either cell death or cell cycle arrest in G1 phase.

### Torin1 inhibits both TORC1 and TORC2 in *S. pombe*

**Fig. 2.**
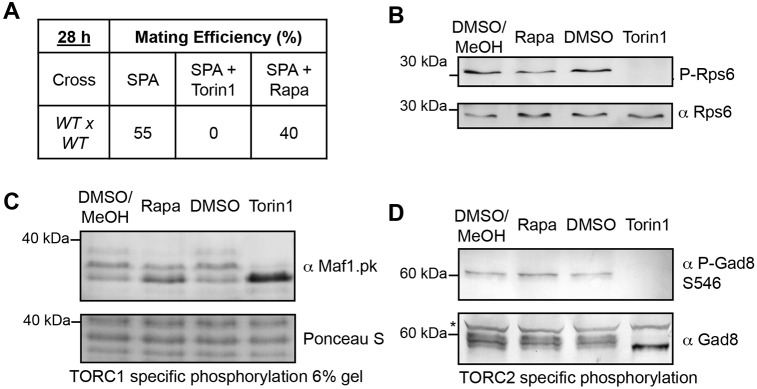
**Torin1 inhibits both TORC1 and TORC2 in *S. pombe*.** (A) Mating efficiency following drug treatment. Cells of opposite mating type were mixed 1∶1 and incubated on SPA plates as indicated (B-D) Wild-type (B,D) and maf1–pk cells (C) were treated with 25 µM Torin1, 300 ng/ml rapamycin or solvent for 30 minutes. Samples were harvested and analysed by western blot using the indicated antibodies. Maf1 phosphorylation is TORC1 specific, Gad8.S546 phosphorylation is TORC2 specific and Rsp6 phosphorylation is regulated by both TORC1 and TORC2.

In fission yeast, both TORC1 and TORC2 signalling is regulated when cells are starved of nitrogen (Matsuo et al., 2007; Murai et al., 2009; Nakase et al., 2013; Nakashima et al., 2012; Petersen and Nurse, 2007; Takahara and Maeda, 2012; Uritani et al., 2006; Weisman and Choder, 2001; Weisman et al., 2007). Cells arrest cell cycle progression in G1 to undergo sexual differentiation and mating (Egel, 2003). Both TORC1 and TORC2 regulate this physiological cell cycle exit and, importantly, TORC2 activity is essential for the G1 arrest. We found that Torin1 completely prevented mating of wild-type cells. In contrast, rapamycin treatment, which only inhibits TORC1, had only a marginal impact on mating proficiency (Fig. 2A). Thus, because Torin1 inhibited growth (which is TORC1-dependent) without inducing a G1 arrest (Fig. 1E) and TORC2 activity is required for G1 arrest, these data suggested that Torin1 inhibited the TOR kinases in both TORC1 and TORC2.

To confirm that Torin1 targets both TOR kinases, we used biochemical read-outs of both TORC1 and TORC2 activity. Phosphorylation of the ribosomal protein S6 (Rps6) is regulated by both TORC1 and TORC2 (Du et al., 2012; Nakashima et al., 2012; Nakashima et al., 2010). In wild-type cells, Rps6 phosphorylation was lost within 30 minutes of Torin1 treatment (Ma et al., 2013), but was only marginally reduced by rapamycin treatment (Fig. 2B). This indicated that Torin1 is targeting TOR. We next monitored the impact of Torin1 addition upon the phosphorylation status of TORC1- and TORC2-specific substrates. Phosphorylation of Maf1, a repressor of RNA polymerase III, is solely dependent on TORC1 activity (Du et al., 2012; Michels et al., 2010), whereas phosphorylation of the AGC kinase Gad8 at serine-546 is uniquely dependent on TORC2 (Matsuo et al., 2003; Tatebe et al., 2010). Maf1 phosphorylation was severely reduced following treatment with Torin1 for 30 minutes (Fig. 2C: note the collapse of the three phosphorylated Maf1 forms (Du et al., 2012) into a single faster-migrating band). Thus, Torin1 inhibited TORC1. Rapamycin also reduced Maf1 phosphorylation, but to a lesser extent, suggesting that rapamycin was a less potent inhibitor of TORC1 than Torin1. Because Ponceau S staining is linear with protein concentration *R*^2^ = 0.99 (Kowalczyk et al., 2013), it was used as loading control for Maf1–pk. Gad8 dephosphorylation was seen after Torin1 addition, whereas rapamycin had no impact upon Gad8 phosphorylation status (Fig. 2D). This suggested that, unlike rapamycin, Torin1 inhibited TORC2. In summary we have shown that, in contrast to rapamycin, Torin1 inhibited TOR in both the TORC1 and TORC2 complexes. Only *tor2^+^* (TORC1 complex) is essential for cell growth (Weisman and Choder, 2001), making it likely that the growth arrest was a consequence of inhibition of TORC1 alone.

### A mutation in the ATP-binding pocket of Tor2 provides Torin1 resistance

**Fig. 3.**
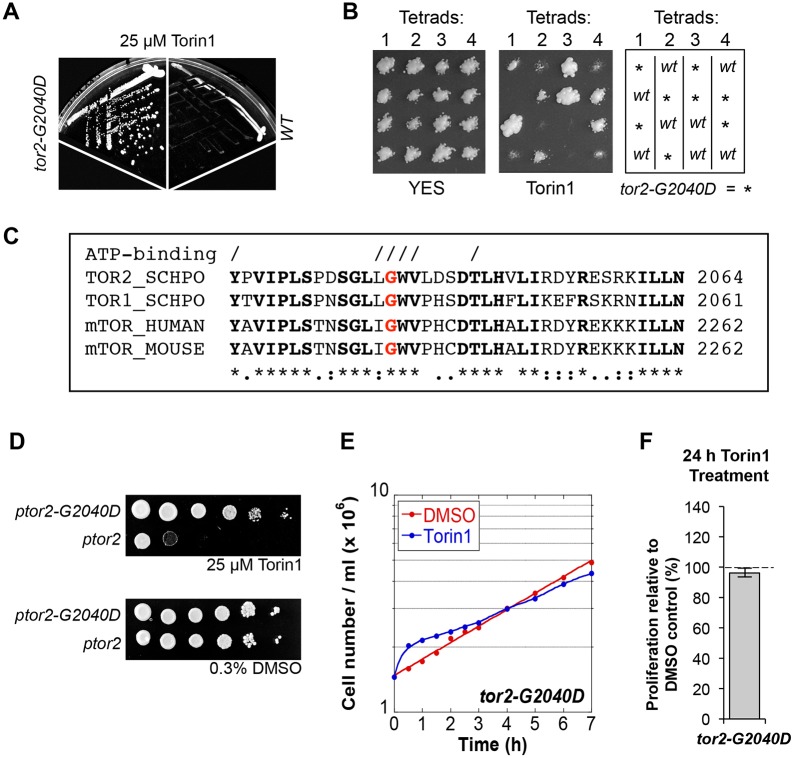
**A mutation in the ATP-binding pocket of Tor2 provides resistance to Torin1.** (A,B) The indicated strains were grown on EMMG plates containing 25 µM Torin1. (B) 2∶2 segregation of Torin1-resistance. Four tetrads were replicated onto YES and YES + Torin1 plates, to the right a diagram illustrates the genotype of the four spores (C) Alignment of TOR kinases; conserved residues are highlighted in bold and residues forming the ATP-binding site are indicated by /. Glycine correspending to *tor2.G2040* is highlighted in red. (D) Expression of *tor2.G2040D* provides resistance to Torin1. (E,F) Cells were treated as in Fig. 1B,C, cell number was counted (E) and proliferation relative to vehicle calculated after 24 hours (F).

We next isolated mutations that allowed cells to grow in the presence of the drug. Following random mutagenesis by exposure to ultraviolet light, cells were plated onto medium containing 25 µM Torin1. A point mutation in the essential *tor2*^+^ gene (TORC1 complex), *tor2-G2040D*, enabled growth in the presence of the drug (Fig. 3A,B). Torin1 resistance segregated with a Mendelian ratio, 2∶2, following mating between wild-type and *tor2-G2040D* cells (Fig. 3B), indicating that Torin1 resistance arose from the *tor2-G2040D* mutation alone. The glycine at position 2040 within the ATP-binding pocket of the kinase domain is conserved throughout eukaryotes (Fig. 3C). To confirm that this mutation in *tor2^+^* did indeed confer Torin1-resistance, plasmids expressing wild-type *tor2*^+^ and *tor2-G2040D* were transformed into a *tor2* deletion strain. Torin1 arrested growth of the strain that expressed the wild-type *tor2^+^* gene, whereas expression of the mutant allele protected cells from this growth arrest (Fig. 3D). The growth rate of *tor2-G2040D* cultures was largely unaffected by exposure to Torin1 (Fig. 3E,F). Together these results indicated that the *tor2-G2040D* mutation provides resistance to Torin1.

### The *tor2-G2040D* mutation alters the dephosphorylation of TORC1 substrates following Torin1 treatment

**Fig. 4.**
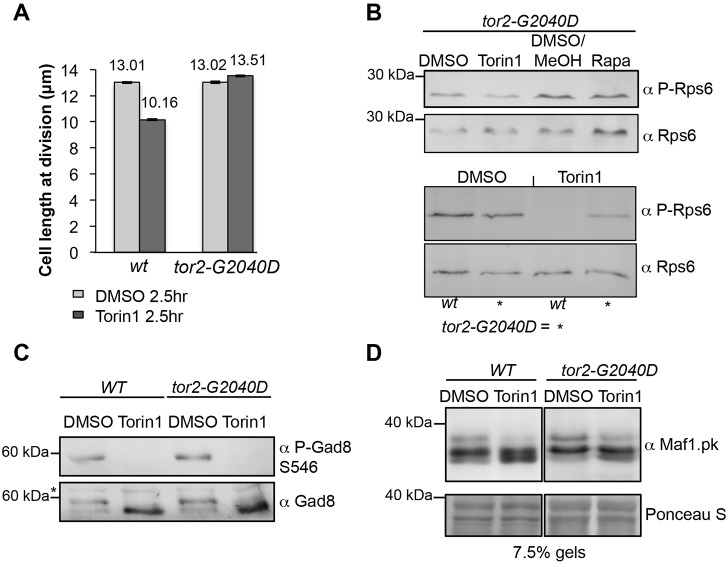
**The *tor2.G2040D* mutation alters the dephosphorylation of TORC1 substrates following Torin1 treatment.** (A) Cell length at division of indicated strains (*n* = 200). After 2.5 hours of Torin1 treatment, 10% of wt cells divide (see Fig. 5A). (B-D) Western blot using the indicated antibodies. Cells were treated as in Fig. 2. (C) Cells were treated with 25 µM Torin1 for 10 minutes. A non-specific band is indicated by an asterisk (*). (D) Cells were treated with 750 nM Torin1 for 10 minutes.

Cell size at division provided further evidence of drug resistance in the *tor2-G2040D* mutant. The reduction in cell size normally induced by treatment with 25 µM Torin1 (Fig. 1F; Fig. 4A) was not observed in *tor2-G2040D* cells (Fig. 4A). Furthermore, biochemical read-outs of TOR activity also revealed resistance. Unlike wild-type cells (Fig. 2B), Rps6 phosphorylation persisted for 30 minutes after drug treatment of *tor2-G2040D* cells (Fig. 4B). In contrast, Gad8 phosphorylation at serine-546 was lost from *tor2-G2040D* cells after Torin1 treatment (Fig. 4C), confirming that Torin1 was still able to inhibit TORC2 in the *tor2-G2040D* (TORC1 complex) mutant. Phosphorylation of Maf1 (a measure of TORC1 activity) was compromised within 10 minutes of the addition of 750 nM Torin1 to wild-type cells (Fig. 4D). In contrast, hyper-phosphorylated forms of Maf1 were still observed in the Torin1-treated *tor2-G2040D* mutant, resulting in less hypo-phosphorylated Maf1 in the *tor2-G2040D* mutant. Together these data suggest that Torin1 had a reduced effect on TORC1 activity in the *tor2-G2040D* mutant. This is expected because Torin1 is an ATP-analogue that is inhibiting an essential kinase, Tor2. If the mutation in the ATP-binding pocket completely blocked Torin1 binding, it would most likely also block ATP-binding to this essential kinase, thereby killing the cell. The resistant mutant is therefore likely to strike a compromise between blocking binding of the ATP analogue (Torin1) and allowing binding of the highly related ATP, to support viability. In summary, mutation of a glycine residue to aspartate in the ATP-binding site of Tor2 provides resistance to the ATP analogue Torin1 and thereby allows cell proliferation in the presence of the drug. This confirms the specificity of Torin1 for TOR kinases in fission yeast. It also validates Torin1 use as a tool for studying TOR function in *S. pombe*.

### Torin1 induces cells to advance into mitosis mainly through TORC1 inhibition

**Fig. 5.**
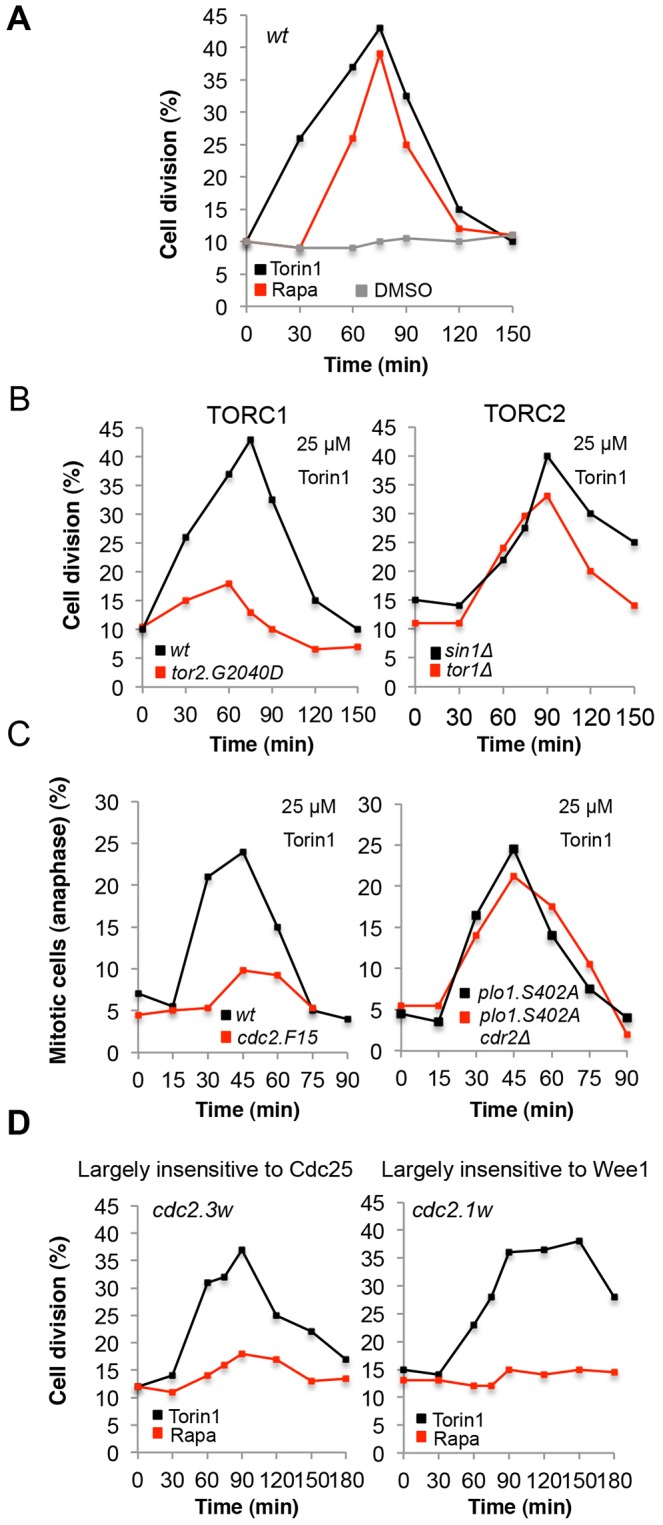
**Torin1 induces cells to advance into mitosis.** (A-D) Cells of the indicated strain were treated with Torin1, rapamycin or DMSO for the indicated times and the percentages of dividing cells (A,B,D) or mitotic cells in anaphase (C) were calculated. Graphs are representative of at least two independent experiments.

*S. pombe* is a rod-shaped cell that grows by tip extension whilst maintaining a constant cell diameter. Cells commit to mitosis and cease growth once a critical length is achieved. Cell length at division is therefore a direct read-out of the time at which cells execute the G2-M transition (Fantes and Nurse, 1977; Nurse and Thuriaux, 1977). This threshold cell length is determined by the environment and correlates with TOR activity (Petersen and Nurse, 2007). When TOR activity is inhibited with either rapamycin or by a decrease in nutrient quality, wild-type cells decrease their size threshold for division and advance into mitosis at a reduced length (Petersen and Nurse, 2007). Consistently, TOR inhibition in wild-type cells by Torin1 lowered the size threshold for mitotic commitment (Fig. 1F; Fig. 4A), leading to a transient burst of mitosis and cell division (Fig. 5A,C). The more rapid response to Torin1 treatment than to rapamycin again implies a more efficient mode of TOR inhibition (Fig. 5A). To determine whether the Torin1-based acceleration of mitosis was elicited through TORC1 or TORC2, cells harbouring mutations in each complex were exposed to the drug (Fig. 5B). TORC1 function was not inhibited by Torin1 in cells expressing *tor2-G2040D* (TORC1 mutant) (Figs 3, 4), which enabled the assessment of the contribution of TORC2 to the mitotic advance in this strain. The Torin1 response of *tor2-G2040D* cells was severely impaired (Fig. 5B), suggesting that Torin1 primarily advances mitosis through inhibition of TORC1. Conversely, mutants in TORC2 (*tor1-Δ*, *sin1-Δ*) both accelerated mitotic commitment, resulting in a peak in the frequency of dividing cells (Fig. 5B). We conclude that the predominant mechanism by which Torin1 advanced mitotic onset was through the inhibition of TORC1.

### Torin1 causes cells to advance into mitosis through the regulators of maturation promoting factor

We have previously shown that mild TOR inhibition with rapamycin also promotes mitotic onset, and that this advance relied upon the mitotic kinase Polo kinase Plo1. Phosphorylation of Plo1.S402 promotes Plo1 recruitment to the spindle pole body (centrosome) to trigger activation of Cdk1–cyclin-B through regulation of Wee1 and Cdc25 (Grallert et al., 2013; Petersen and Hagan, 2005). The Wee1 kinase inhibits Cdk1–cyclin-B through phosphorylation of tyrosine 15 within the active site of Cdc2 (Featherstone and Russell, 1991; Gould and Nurse, 1989), and Cdc25 phosphatase removes the inhibitory phosphate placed on Cdc2 by Wee1 (Millar et al., 1991). Combined regulation of both Cdc25 and Wee1 is essential for this weak rapamycin-induced TORC1 inhibition to promote Cdk1–cyclin-B activation (Petersen and Nurse, 2007). Interestingly, the acceleration of mitosis that is triggered by Torin1 treatment persists in the phosphorylation-refractory *plo1.S402A* allele (Fig. 5C), indicating that, following the stronger Torin1-induced TORC1 inhibition, either *plo1.S402A*-independent activation of Cdc25 or inhibition of Wee1 alone is sufficient to advance mitotic onset. We therefore assessed the impact of Torin1 treatment on the Cdc2 mutants *cdc2-1w* and *cdc2-3w* (Fantes, 1981; Thuriaux et al., 1978). *cdc2-1w* is largely insensitive to Wee1, such that Cdk1–cyclin-B activity is mainly regulated by Cdc25 function. In contrast, *cdc2-3w* is largely unresponsive to Cdc25, and hence Cdk1–cyclin-B activity is mainly dependent upon Wee1 control in this mutant. In contrast to mild TOR inhibition with rapamycin, where neither *cdc2.1w* nor *cdc2.3w* advanced mitosis, both mutants advanced mitotic commitment following addition of Torin1 (Fig. 5D) (Petersen and Nurse, 2007). Together, these findings indicate that Torin1 very efficiently activates Cdc25 and inhibits Wee1, and either activation of Cdc25 or inhibition of Wee1 activates Cdk1–cyclin-B to a sufficient degree to advance the timing of mitotic commitment. Importantly, the acute Torin1-induced mitotic advance was regulated through Cdc2 tyrosine 15, because the *cdc2.F15* mutant, which cannot be regulated by either Wee1 or Cdc25 on Tyr15, did not efficiently advance mitosis in response to Torin1 treatment (Fig. 5C).

### Torin1 induces a decline in Wee1 levels and a reduced Cdc25 migration

**Fig. 6.**
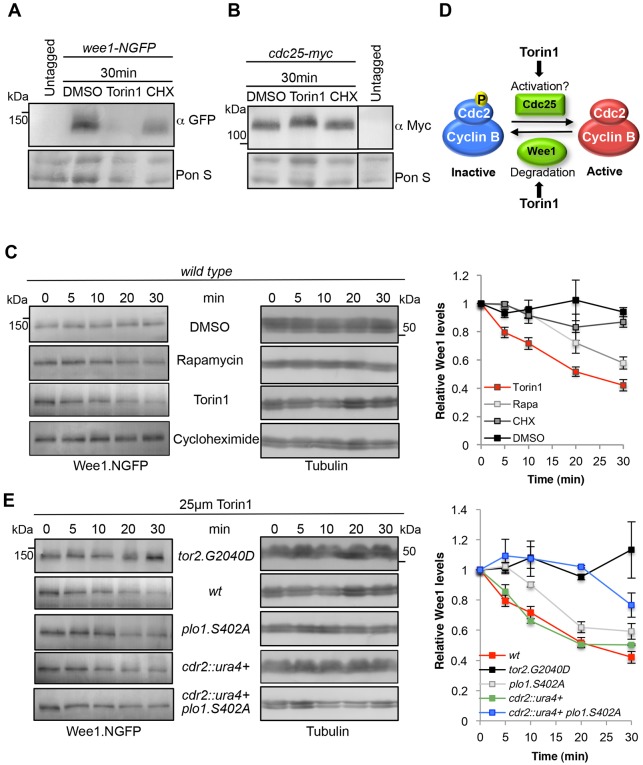
**Torin1 induces a Plo1- and Cdr2-controlled decline in Wee1 levels.** (A,B,C,E) Cells were treated with Torin1, cycloheximide (CHX), rapamycin or DMSO for indicated times. (A,B) Western blot of Cdc25 or Wee1. (D) Inhibition of TORC1 by Torin1 in wild-type cells traps the Cdk1–cyclin-B complex in its active dephosphorylated form as Wee1 levels are reduced and Cdc25 is modified. (C,E) Western blots of Wee1 levels and quantification of Wee1 levels from 3 individual experiements. Torin1-treated wt cells are shown twice for comparison.

TOR inhibition promotes Sty1 activation, which in turn leads to Plo1 recruitment to the spindle pole bodies to trigger activation of Cdk1–cyclin-B. This is regulated through Wee1 and Cdc25 (Grallert et al., 2013; Petersen and Hagan, 2005). However, it is unclear how Wee1 and Cdc25 activities are controlled. Interestingly, the sharp rise in the mitotic index following Torin1 addition to the culture coincided with a decline in Wee1 levels (Fig. 6A,D,E). Wee1 is tagged with GFP at the N-terminus, and this strain has a wild-type response to Torin1 treatment (data not shown). The degree to which Torin1 addition reduced Wee1 levels exceeded that seen upon inhibition of global protein synthesis by cycloheximide treatment and that of mild TOR inhibition with rapamycin, indicating that Wee1 turnover had been actively promoted by the inhibition of TOR function (Fig. 6A,C). Importantly, this decline in Wee1 was not observed in the *tor2.G2040D* mutant (Fig. 6E). In addition, the decline in mobility of Cdc25 in SDS-PAGE that accompanies its activation (Kovelman and Russell, 1996; Moreno et al., 1990) was seen within 30 minutes of Torin1 treatment (Fig. 6B). We suggest that inhibition of TORC1 by Torin1 in wild-type cells traps the Cdk1–cyclin-B complex in its active dephosphorylated form (Fig. 6D) to promote mitotic commitment at a reduced cell size (Fig. 4A; Fig. 5C). The molecular basis for these multiple controls of the Cdk1–cyclin-B complex in response to TOR inhibition is very interesting – here we focused our attention upon the regulation of Wee1.

### Torin1 induces a Plo1- and Cdr2-controlled decline in Wee1 levels

It has previously been shown that Plo1 regulates Wee1 activity, because the enhanced Plo1 recruitment to the spindle pole body by Cut12 is sufficient to overcome the lack of Cdc25 (Hagan, 2008). The decline in Wee1 levels was delayed, but it was still reduced in the *plo1.S402A* mutants after Torin1 treatment (Fig. 6E). This suggested that Wee1 could also be downregulated through a *plo.S402A*-independent mechanism following Torin1-induced TOR inhibition. SAD (synapses of amphids defective) family kinases have been shown to also regulate zWee1 levels (Müller et al., 2010; Young and Fantes, 1987). We therefore asked whether the fission yeast Cdr2 (SAD kinase) along with Plo1 were responsible for the changes in Wee1 levels that arise from Torin1 inhibition. The Torin1-induced decline of Wee1 was severely compromised by the absence of combined Cdr2 and Plo1 function, indicating that TORC1 control of Wee1 turnover was regulated by both kinases but independently (Fig. 6E). The acceleration of mitosis that is triggered by Torin1 persisted in the *plo1.S402A cdr2Δ* double mutant (Fig. 5C), which again demonstrated that regulation of Cdc25 only was sufficient to advance mitotic onset following the strong Torin1-induced TOR inhibition. How Plo1 and Cdr2 independently control this novel Torin1-induced Wee1 turnover is unclear at present. However, Wee1 resembles its budding yeast counterpart Swe1p (Harvey et al., 2005; Sakchaisri et al., 2004) in being very heavily phosphorylated (Y. D. Tay and I. Hagan, personal communication), and Cdr2 has previously been shown to control phosphorylation of Wee1 (Kanoh and Russell, 1998), suggesting that either kinase could directly modulate Wee1 phosphorylation to control protein levels.

### mTORC1 inhibition reduces Wee1 levels

**Fig. 7.**
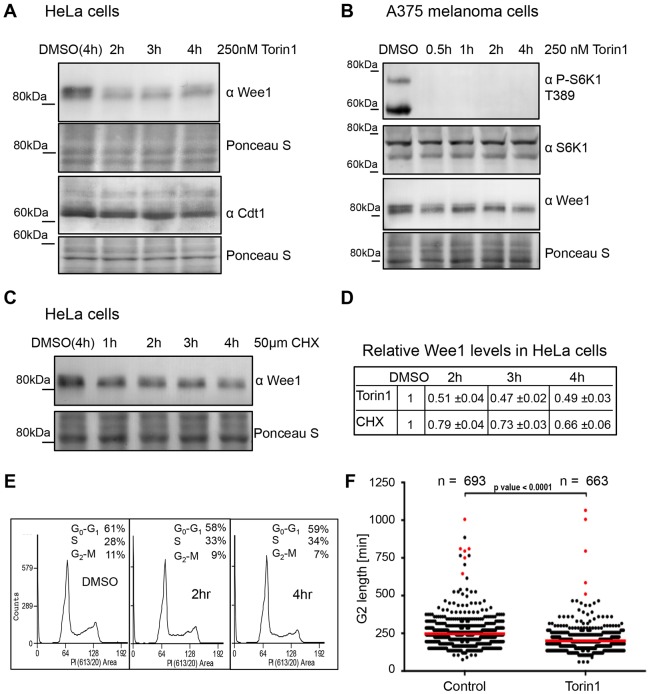
**mTORC1 inhibition advances mitosis, as Wee1 is lost.** (A) HeLa cells were treated with 250 nM Torin1 and samples harvested at the indicated time points and analysed by western blot using the indicated antibodies. (B) A375 cells were treated with 250 nM Torin1 and analysed by western blot. (C) Cycloheximide (CHX) treatment of HeLa cells. (D) Quantification of A and C. (E) HeLa cells were treated with 250 nM Torin1 and DNA content was analysed by flow cytometry. (F) HeLa Kyoto cyclin-B1–Venus^−/+^ mRuby–PCNA^−/+^ H3.3–CFP cells were treated with 250 nM Torin1 or solvent (DMSO). Cell cycle progression was analysed by time-lapse microscopy using mRuby–PCNA marker. G2 length in individual cells was measured from the time point when the PCNA foci form. Red lines are mean values of four independent experiments; red dots represent cells that were in G2 phase until the end of experiment.

Torin1-induced inhibition of mTOR in mammalian cells limits protein synthesis and arrests cells in G1 at a reduced cell size (Thoreen et al., 2009). However, it is unclear whether cells also advance mitotic commitment before this growth arrest. The link between TOR signalling and Wee1 control in fission yeast prompted us to ask whether mTOR similarly controls Wee1 levels in human cells. Initial experiments using synchronization approaches, which are widely used to manipulate cell cycle progression in mammalian cells, revealed a small decrease in Wee1 levels (data not shown). However, it is possible that synchronization invoked stress-induced MAPK signalling that could have impacted on mTOR signalling prior to drug treatment (Hernández et al., 2011; Ma et al., 2005). We therefore turned to asynchronous populations; it was noticeable that Wee1 levels in unperturbed cell cultures were markedly reduced after 2 hours of mTOR inhibition with Torin1. This reduction in Wee1 levels was seen in both HeLa cells and in A375 melanoma cells (Fig. 7A,B). Importantly cycloheximide treatment did not promote the same degree of Wee1 instability (Fig. 7C,D). Wee1 levels have been reported to be low in the G1 phase of the cell cycle (Watanabe et al., 2005), therefore this rapid Torin1-induced decline in Wee1 levels could simply reflect a rapid change in the cell cycle profile of the population. However, flow cytometry analysis and the level of the G1-specific marker Cdt1 provided no evidence for an increase in the G1 population at these early time points (Fig. 7A,E). Thus, these experiments in mammalian cells mirrored the results from fission yeast in that mTOR inhibition led to reduced Wee1 levels.

### mTOR inhibition advances mitotic onset

We next addressed the possibility that mTOR control of Wee1 levels would advance mitotic commitment by shortening the G2 phase of the cell cycle. We used a non-invasive live-imaging assay to monitor the impact of Torin1 inhibition of mTOR activity on HeLa cells. We defined the start of G2 phase as the loss of the last foci of the DNA replication marker proliferating cell nuclear antigen (PCNA), and the end of G2 phase as nuclear envelope breakdown, when nuclear PCNA dispersed throughout the cytoplasm. Torin1 treatment accelerated the mitotic commitment of HeLa cells, as indicated by the finding that the duration of G2 phase contracted from 247 minutes to 200 minutes (Fig. 7F). This 20% decrease in the duration of G2 phase in HeLa cells was highly reminiscent of previous observations in fission yeast, in which TOR inhibition in synchronous fission yeast cultures reduced the G2 phase by 25% (Petersen and Nurse, 2007). Together, these findings provide the first demonstration that, just like in fission yeast, inhibition of mTOR can advance mitosis in mammalian cells.

## DISCUSSION

We show that complete inhibition of TORC1 function by the addition of the ATP analogue Torin1 blocks cell proliferation of fission yeast. Because the signalling pathways upstream of TORC1 are conserved between this yeast and humans, we anticipate that the use of Torin1 in *S. pombe* will make a significant contribution to our understanding of the architecture and control of mTOR signalling in mammals.

**Fig. 8.**
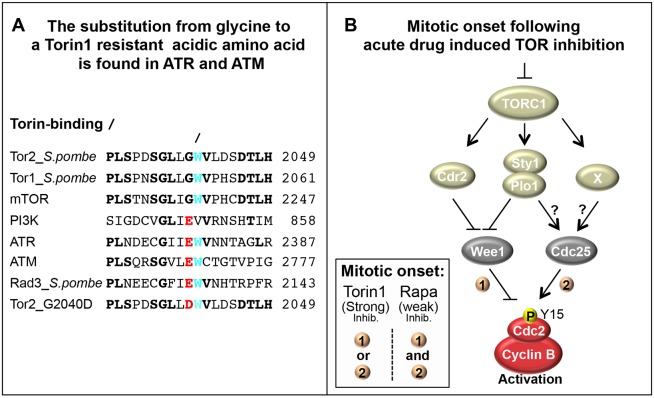
**The convergence of multiple pathways to control Cdc2–cyclin-B activity.** (A) Alignment of TOR and TOR-related kinases. Conserved residues are highlighted in bold. The key Torin-interacting tryptophan is shown in cyan. The Torin1 resistant *tor2-G2040D* is shown in red. (B) A model suggesting that when Torin1 inhibits TORC1, Wee1 levels decline. This traps Cdc2–cyclin-B in its active conformation, driving entry into mitosis at a reduced cell size. The presence of molecule X is implied by advanced mitosis in torin1-treated *plo1.S402A cdr2::ura4^+^* double mutants. Insert: in contrast to rapamycin, regulation of either Wee1 (1) or Cdc25 (2) is sufficient to advance mitotic onset following the strong Torin1-induced TOR inhibition.

A single glycine-to-aspartate mutation at position 2040 of the essential *S. pombe* kinase Tor2 conferred Torin1 resistance, providing the first *in vivo* validation of the specificity of Torin1 for TOR kinases in any eukaryote (Figs 3,4). The site of this mutation within the ATP-binding pocket is conserved in mTOR (Fig. 3C). Torin1 docking in the ATP-binding pocket of mTOR (Liu et al., 2012) is proposed to occur through a putative hydrogen bond between Torin1 and Val2240. Furthermore, the recently identified crystal structure of mTOR in complex with the second-generation Torin2 inhibitor places Ile2237 and Trp2239 within 4 Å of the tricyclic benzonaphthyridinone ring (shared with Torin1) (Yang et al., 2013). In fact, the pivotal interaction between Torin2 and mTOR is through the Trp2239 residue, which interacts with ten atoms from the ring moiety of Torin2 and is found directly adjacent to the glycine [equivalent to the site of mutation in fission yeast Tor2 (Fig. 8A)]. Torin1 and Torin2 both inhibit mTOR with an 800-fold greater specificity over PI3Ks (Liu et al., 2013; Thoreen et al., 2009). Importantly, PI3Ks do not contain the key Torin-interacting tryptophan (Fig. 8A) (Yang et al., 2013). It was recently shown that Torin2 has a 10-fold lower affinity for the two PI3K-like kinases ATM and ATR (Liu et al., 2013). Interestingly, ATM, ATR and the fission yeast homologue Rad3 all contain the key tryptophan residue (Fig. 8A). However, they mimic the Torin1 resistant *tor2* mutant that we have isolated here in having a charged glutamic acid instead of the glycine found at the site of the *tor2* mutation in TOR kinases (Fig. 8A) – this is likely to explain the reduced drug potency towards these closely related kinases. The identification of this conserved site has implications for studies in mammalian cells, where this mutant will be useful in eliminating the possible off-target effects.

Here we find that the TORC1 control of mitotic entry and cell size at division can be achieved by the convergence of multiple independent pathways that regulate Wee1 and Cdc25 to control Cdc2–cyclin-B activity (Fig. 8B). Importantly, both rapamycin- and Torin1-stimulated Cdc2–cyclin-B activation is regulated through Wee1 and Cdc25 to control Cdc2.Y15 phosphorylation. However, the level of Cdc2–cyclin-B activation varied depending upon the degree of TOR inhibition. Under mild TOR inhibition with rapamycin, the advance of mitotic commitment required simultaneous control of both Cdc25 and Wee1 (Fig. 8B). In contrast, the enhanced level of TORC1 inhibition arising from the use of Torin1 did not rely upon dual control of both of these regulators – rather a singular impact upon either Cdc25 or Wee1 activity alone was sufficient to advance mitotic commitment (Fig. 8B). Importantly, we find that the reduction in Wee1 levels upon acute TORC1 inhibition in *S. pombe* is also seen upon mTORC1 inhibition in human cells (Figs 6, 7), revealing a novel, universal aspect of TOR signalling. Furthermore, the Torin1-induced advancement of mitotic commitment that we observed in HeLa cells (Fig. 7F) is consistent with the notion that the mTOR kinase controls mitotic commitment.

Interestingly, in pilot experiments, no advancement in the timing of mitosis was observed in retinal pigment epithelium (RPE) cell lines following Torin1 treatment (data not shown). Therefore, Torin1-induced advanced mitotic commitment is likely to be cell line and context specific. However, in RPE cells, TOR signalling appears to function differently because TORC2-dependent activation of AKT1 is absolutely required for its activity (Parrales et al., 2013), whereas this is not the case in other cell lines. Furthermore, HeLa cells are p53 deficient. They have reduced PTEN levels, and therefore have increased TOR signalling (Feng, 2010). In addition, because p53 also impacts on TOR signalling through the tuberous sclerosis (TSC) and AMP-activated protein kinase (AMPK) TOR inhibitors (Feng, 2010), the steady-state TOR activity in RPE and HeLa cell lines is likely to be very different. This difference is likely to account for the different impacts of Torin1 inhibition in the two cell lines.

In summary, mammalian cells can exploit the same types of TOR-mediated control of Cdk1–cyclin-B activity that couple changes in the timing of mitotic commitment to environmental cues in fission yeast, as both HeLa and A375 cell lines reduced Wee1 levels following mTOR inhibition with Torin1. The ability of Torin1 to reduce cell growth and proliferation has made it an attractive anti-cancer drug candidate. Similar levels of excitement have been provoked by the success of a Wee1 inhibitor in pre-clinical models in which Wee1 inhibition promoted a lethal mitotic catastrophe in cancer cells whose DNA integrity was compromised (Kreahling et al., 2012). We therefore provide a novel means by which Torin1-mediated inhibition of TOR activity could offer therapeutic benefit in particular tumour contexts.

## MATERIALS AND METHODS

### Yeast cell culture and reagents

The *S. pombe* strains used in this study are listed in supplementary material Table S1. Unless otherwise stated, cells were grown at 28°C in Edinburgh minimal medium 2 (EMM2-N ForMedium) supplemented with 20 mM glutamate (EMMG). Cells were grown exponentially at 28°C for 48 hours. For growth on solid media, cells were grown at 30°C on plates containing YES or EMMG.

### Molecular biology

QuikChange (Stratagene) site-directed mutagenesis was used to generate the plasmid containing *tor2-G2040D* (forward primer: 5′-CCTTTTAGACTGGGTTTTGGATAGCGATAC-3′, reverse primer: 5′-CAAAACCCAGTCTAAAAGGCCTGAATCCGGTG-3′). Wild-type *tor2^+^* and *tor2-G2040D* were subsequently cloned into the pREP42–NAT vector (kind gift from I. Hagan). Plasmids were transformed into *S. pombe* using lithium acetate (Bähler et al., 1998).

### Growth assays

Torin1 was synthesised at the CRUK Manchester Research Institute according to the published protocol (Liu et al., 2010), yielding a product with >95% purity as determined by liquid chromatography mass spectrometry and proton NMR. Torin1 was stored as a solid in the dark and dissolved at 50°C in DMSO (Sigma-Aldrich) immediately before use, with a stock concentration of 7.5 mM. For cell growth assays, cells were grown exponentially for 48 hours to 2.5×10^6^ cells/ml before treatment with Torin1 at a final concentration of 25 µM or equivalent DMSO vehicle control. Growth at 28°C was monitored by optical density (OD) at 595 nm. For cell survival assays, following treatment with Torin1 or DMSO as above, 500 cells were spread on YES plates in duplicate. Colonies were counted after incubation at 30°C. For growth on solid media 2.5×10^6^ cells/ml were spotted on plates containing EMMG + Torin1 (25 µM final) or DMSO (0.33% final v/v), or EMMG + rapamycin (300 ng/ml final from 2 mg/ml stock; Sigma-Aldrich) or 1∶1 DMSO∶MeOH (0.15% final v/v). For protein turnover assays, 100 µg/ml cycloheximide was used.

### Torin1 screen

Wild-type cells were grown to 2.5×10^6^ cells/ml. 100,000 cells/plate were plated onto 10 EMMG plates containing 25 µM Torin1 and were exposed to 0.015 J of UV irradiation per plate using a UV lamp cabinet (Uvitec, Cambridge). Plates were incubated at 30°C for 3 to 5 days. Isolated mutants were streaked to single colonies on YES plates and replica-plated to EMMG plates containing 25 µM Torin1 to remove false positives. Three candidates were backcrossed a minimum of three times before genomic DNA was isolated (Qiagen Gentra Puregene Yeast/Bact. Kit) and sequenced by whole genome sequencing (GATC Biotech, Konstanz, Germany).

### Flow cytometry

*S. pombe* DNA content and cell size were measured by flow cytometry as previously described (Costello et al., 1986). Samples were sonicated and 30,000 events processed using a Beckman Coulter Cyan ADP instrument with 488 nm excitation detection filter and 530/40 nm bandpass. HeLa cells were fixed drop-wise with 70% ethanol and 5000 events processed as above. Data were analysed using Summit 4.3 software (Beckman Coulter).

### Protein extraction and western blotting

For western blotting of *S. pombe* cells, 6×10^7^ cells were fixed and total protein extracts were prepared by precipitation with trichloroacetic acid (TCA) as previously described (Caspari et al., 2000). HeLa and A375 cells were lysed in SDS sample buffer [60 mM Tris-HCl (pH 6.8), 10% glycerol, 1% SDS, 2% β-mercaptoethanol, 0.01% Bromophenol Blue]. Following gel electrophoresis, proteins were transferred onto a polyvinylidene difluoroide (PVDF) membrane (Millipore), blocked in TBS plus 3% dried milk and incubated with primary antibodies in TBS plus 3% milk overnight at 4°C. After washing (4×30 minutes with TBS plus 0.05% Tween20), membranes were probed with secondary antibodies linked to alkaline phosphatase (Sigma-Aldrich). Membranes were developed after washing as above by addition of substrate: nitro-blue tetrazolium chloride (NBT; VWR International)/5-bromo-4-chloro-3′-indolylphosphate p-toluidine (BCIP; Molekula) in AP buffer (100 mM NaCl, 5 mM MgCl_2_, 100 mM Tris-HCl pH 9.5). The following primary antibodies were used in this study: mouse anti-phospho-(Ser/Thr) Akt substrate (PAS) antibody to detect phospho-Rps6 (1∶2000; Cell Signaling Technology), mouse anti-S6 antibody to detect total Rps6 (1∶2000; Abcam), mouse anti-V5-Tag antibody to detect Maf1–pk (1∶1000; AbD Serotec), rabbit anti-phospho-Gad8 S546 antibody to detect phospho-Gad8 S546 (1∶500; raised by Eurogentec) (Du et al., 2012), sheep anti-Gad8 antibody to detect total Gad8 (1∶200; raised by Scottish National Blood Transfusion Service, Midlothian) (Du et al., 2012), mouse anti-myc antibody to detect Cdc25–myc (1∶2000; Millipore), mouse anti-GFP antibody to detect Wee1–GFP (1∶100; Roche), rabbit anti-Wee1 antibody to detect human Wee1 (1∶500; New England Biolabs), rabbit anti-Cdt1 antibody to detect Cdt1 (1∶1000; New England Biolabs).

### Mating assays

Cells were grown exponentially for 24 hours in YES at 28°C to a concentration of 2.4×10^6^ cells/ml. 4×10^6^ cells were mixed with an equal number of wild-type cells (h^+^/h^−^), washed in sterile water and spotted onto sporulation agar (SPA) plates or SPA plates supplemented with Torin1 (25 µM final) or rapamycin (300 ng/ml final). Plates were incubated at 30°C for 28 hours before 200–500 cells or zygotes were counted.

### Spot tests

Cells were grown exponentially for 24 hours only in YES at 28°C to 2.4×10^6^ cells/ml. A tenfold dilution series starting with 1×10^6^ cells was spotted onto YES plates or EMMG plates supplemented with 25 µM Torin1 or 0.33% DMSO. Plates were incubated at 30°C and growth was monitored daily for 4 days.

### Fluorescence microscopy

Cells were treated as for liquid growth assays and samples fixed with 30% formaldehyde (10% final v/v) for fluorescence microscopy every 15–60 minutes for 3 hours. Septa were stained with calcofluor white (Sigma-Aldrich) and cells were observed under a fluorescence microscope at 100× magnification. At least 200 cells were counted per time-point. Combined calcoflour/DAPI staining was used to determine the anaphase population; only bi-nucleate non-septating cells were counted as anaphase cells.

### Cell culture

HeLa Kyoto cyclin-B1–Venus^−/+^ mRuby–PCNA^−/+^ H3.3–CFP cells were grown in Advanced DMEM (Gibco) supplemented with 2% FBS (Gibco). A375 cells were grown in DMEM (Gibco) supplemented with 10% FBS (PAA). For protein turnover assays, 50 µM cycloheximide was used.

### Single cell live microscopy

HeLa Kyoto cyclin-B1–Venus^−/+^ mRuby–PCNA^−/+^ H3.3–CFP cells were grown on a 96-well plate (μClear, Greiner). Culture medium was replaced with Leibovitz's L-15 medium (Invitrogen) supplemented with 10% FBS prior to filming. Live-cell imaging was performed using an ImageXpress high-throughput microscope (Molecular Devices). Cells were filmed every 15 minutes for the total duration of 18–24 hours with a ×20 objective. Four to six positions were acquired per well. Exposure time was 200 milliseconds for the mRuby filter.

## Supplementary Material

Supplementary Material
