# Supplementary material for: Torin1-mediated TOR kinase inhibition reduces Wee1 levels and advances mitotic commitment in fission yeast and HeLa cells
Source: J Cell Sci. 2014 Mar 15;127(6):1346–56. doi: 10.1242/jcs.146373 (PMC3953821; doi:10.1242/jcs.146373)
Supplement: Supplementary Material [file supp_127_6_1346__index.html]

Torin1-mediated TOR kinase inhibition reduces Wee1 levels and advances mitotic commitment in fission yeast and HeLa cells — Supplementary Material 

# Torin1-mediated TOR kinase inhibition reduces Wee1 levels and advances mitotic commitment in fission yeast and HeLa cells

## JCS146373 Supplementary Material

**Files in this Data Supplement:**

- **Supplementary Material**
