# Supplementary material for: Torin1-mediated TOR kinase inhibition reduces Wee1 levels and advances mitotic commitment in fission yeast and HeLa cells
Source: J Cell Sci. 2014 Mar 15;127(6):1346–56. doi: 10.1242/jcs.146373 (PMC3953821; doi:10.1242/jcs.146373)
Supplement: Supplementary Material [file supp_127.6.1346_JCS146373.pdf]

Table S1: *S. pombe* strains used in this study

Strain	Genotype	Source
JP3	*h⁻ 972*	Lab stock
JP57	*h⁻ plo1.S402A*	Lab stock
JP350	*h⁺*	Lab stock
JP567	*h⁻ cdc2-1w*	(Thuriaux et al., 1978)
JP568	*h⁻ cdc2-3w*	(Fantes, 1981)
JP1377	*sin1::kanMx6*	*sin1::kanMx6* from (Ikeda et al., 2008)
JP1491	*tor1::ura4⁺ ura4-D18*	*tor1::ura4⁺* from (Matsuo et al., 2003)
JP2337	*cdr2::ura4⁺ ura4-D18*	*cdr2::ura4⁺* from (Bimbo et al., 2005)
JP1662	*h⁺ nmt81-GFPwee1*	(Moseley et al., 2009)
JP1670	*h⁺cdc25:12myc-ura4⁺ ura4-D18*	*cdc25:12myc.ura4⁺* from (Lopez-Girona et al., 1999)
JP1669	*h⁺ tor2-G2040D*	This study
JP1731	*h⁻ maf1pk-kanMx6*	Lab stock
JP1763	*h⁻maf1pk-kanMx6 tor2-G2040D*	This study
JP2081	*tor2-G2040D nmt81-GFPwee1*	This study
JP2089	*h⁺ tor2::ura4+ ura4-d18 leu1-32 pREP42NAT-tor2*	This study *tor2::ura4*+ from (Weisman et al., 2007)

JP2090	*h$^+$ tor2::ura4+ ura4-d18 leu1-32 pREP42NAT-tor2-G2040D*	This study
JP2177	*cdr2::ura4$^+$ nmt81-GFPwee1 ura4-d18*	This study
JP2377	*plo1.S402A nmt81-GFPwee1*	This study
JP2363	*h$^+$ cdr2::ura4$^+$ plo1.S402A ura4.D18*	This study
JP2405	*cdr2::ura4$^+$ plo1.S402A nmt81-GFPwee1*	This study
JP2411	*h$^-$ cdc2::ura4$^+$ leu1.32 ura4.D18 mns21L-cdc2.F15 integrated*	Paul Nurse D832

References

Bimbo, A., Y. Jia, S.L. Poh, R.K. Karuturi, N. den Elzen, X. Peng, L. Zheng, M. O'Connell, E.T. Liu, M.K. Balasubramanian, and J. Liu. 2005. Systematic deletion analysis of fission yeast protein kinases. *Eukaryotic cell*. 4:799-813.

Fantes, P.A. 1981. ISOLATION OF CELL-SIZE MUTANTS OF A FISSION YEAST BY A NEW SELECTIVE METHOD - CHARACTERIZATION OF MUTANTS AND IMPLICATIONS FOR DIVISION CONTROL MECHANISMS. *Journal of Bacteriology*. 146:746-754.

Ikeda, K., S. Morigasaki, H. Tatebe, F. Tamanoi, and K. Shiozaki. 2008. Fission yeast TOR complex 2 activates the AGC-family Gad8 kinase essential for stress resistance and cell cycle control. *Cell Cycle*. 7:358-364.

Lopez-Girona, A., B. Furnari, O. Mondesert, and P. Russell. 1999. Nuclear localization of Cdc25 is regulated by DNA damage and a 14-3-3 protein. *Nature*. 397:172-175.

Matsuo, T., Y. Kubo, Y. Watanabe, and M. Yamamoto. 2003. Schizosaccharomyces pombe AGC family kinase Gad8p forms a conserved signaling module with TOR and PDK1-like kinases. *Embo Journal*. 22:3073-3083.

Moseley, J.B., A. Mayeux, A. Paoletti, and P. Nurse. 2009. A spatial gradient coordinates cell size and mitotic entry in fission yeast. *Nature*. 459:857-U858.

Thuriaux, P., P. Nurse, and B. Carter. 1978. MUTANTS ALTERED IN CONTROL COORDINATING CELL-DIVISION WITH CELL-GROWTH IN FISSION YEAST

SCHIZOSACCHAROMYCES-POMBE. *Molecular & General Genetics*. 161:215-220.

Weisman, R., I. Roitburg, M. Schonbrun, R. Harari, and M. Kupiec. 2007. Opposite effects of Tor1 and Tor2 on nitrogen starvation responses in fission yeast. *Genetics*. 175:1153-1162.
